# Delayed drug-induced catatonia in an adolescent girl—clinical implications: a case report

**DOI:** 10.1186/s13256-024-04819-2

**Published:** 2024-11-12

**Authors:** Max Winerdal, Konstantinos Skordas, Anna Karin Lidehäll, Carin Wilhelmsdotter, Helena Strömbergsson

**Affiliations:** 1 Division of Child and adolescent psychiatry, Uppsala Academic Hospital, Uppsala, Sweden; 2 Department of Medical Sciences, Psychiatry, Uppsala University, Akademiska Sjukhuset, Ingång 10, 751 85 Uppsala, Sweden

**Keywords:** Case report, Catatonia, Mephedrone, Ethyl chloride, Memantine, Bath salt drugs

## Abstract

**Background:**

Catatonia is a potentially life-threatening condition that is characterized by psychiatric and motor disturbances, such as negativism, hypomotility, bradykinesia, and unusual movements. The diagnosis is based on clinical examination and occurs in both pediatric and adult patients and is associated with an increased mortality. Catatonia is associated with psychiatric illnesses such as schizophrenia, major depression, encephalitis, and bipolar disorder. The physiopathology of catatonia is complex and not fully understood. There is an ongoing debate in the medical community whether catatonia is an independent syndrome, or secondary to other mental illnesses. This case presentation is unique, as there are few reports describing cases of isolated catatonic syndrome in the absence of any other psychiatric or medical condition with a delayed onset caused by recreational drug abuse.

**Case presentation:**

We present the case of a 17-year-old Caucasian athletic girl with no previous contact with child and adolescent psychiatry, nor any previous drug abuse. After recreational intake of drugs, there was a delay of approximately 7 days, before the patient searched care with symptoms that were at a later stage recognized as catatonia. Treatment with a high dose of lorazepam in combination with memantine and lithium resulted in a regression of the symptoms. After 6 weeks the patient could be discharged from the hospital almost fully recovered.

**Conclusions:**

An acute onset of psychomotor symptoms without any previous history of mental illnesses must be addressed early as a potential catatonic syndrome. Delayed onset of catatonic symptoms after intake of drugs should not be overlooked, and we here suggest that mephedrone might be capable of inducing delayed catatonia. It is feasible to use memantine as an adjuvant to the treatment of catatonia in adolescents.

## Background

We here present a case of drug-induced catatonia in an adolescent Caucasian patient with heredity for bipolar disorder after supposed intake of “bath salts” and ethyl chloride. Mephedrone, the major constituent in “bath salts”, inhibits monoamine reuptake and induces release. A massive increase of serotoninergic levels has been reported, as well as increased levels of dopamine, glutamate, and norepinephrine [1], which speculatively could induce sufficient receptor adaptation, a prerequisite for delayed onset of withdrawal reactions. Catatonia is a severely debilitating disorder characterized by symptoms of negativism, mutism, stupor, excitement, repeated stereotyped movements, staring, and grimacing [Diagnostic and Statistical Manual of Mental Disorders (DSM)]. The condition can occur during the whole life span. In pediatric patients, catatonia has traditionally been associated with psychiatric illnesses such as schizophrenia, major depression, encephalitis, and bipolar disorder [2]. During recent years, it has been increasingly recognized that catatonia in pediatric patients is associated with neurodevelopmental disorders, such as autism spectrum disorders and intellectual disability [3]. Catatonia may also appear without any prior psychiatric comorbidity in the presence of medical and neurological illness, or as a result of prior intake of drugs, both prescribed [4] and illicit [1]. The condition is rare, with a prevalence of 0.4% in adolescent patients admitted to psychiatric in-patient clinics [5]. The large variation in clinical manifestation makes recognition of catatonia challenging, and in pediatric patients the condition is most likely underdiagnosed. Catatonia may develop into malignant catatonia, which is a life-threatening condition. The physiopathology of catatonia is complex and not fully understood. At the molecular level, theories include hypofunction of GABA-A receptors, dysregulated dopamine signaling, and NMDA receptor activity resulting in NMDA hyperactivity in the striato-cortical or the cortico-cortical pathways [6]. This case report is, to our knowledge, unique in its description of a possibly drug-induced delayed onset of catatonic signs and symptoms without previous psychiatric illness.

### Case presentation

A 17-year-old Caucasian female high school student with no previous psychiatric history was brought to the emergency department. Five days prior to seeking emergency care, the patient had returned from a journey to the Alps. During the journey, the patient had been exposed to drugs in the form of tablets and spray that smelt of chloride. At the time of exposure, the patient reacted to the drugs with confusion and hallucinations. In the aftermath, within 24 hours of exposure, the acute symptoms were replaced with strong anxiety and signs of depression. The police suggested that the drugs were most likely “bath salts” and “ethyl chloride”. The patient sought emergency care due to confusion, dissociations, insomnia, anxiety, mydriasis, and behavioral changes. On admission, it was noted during the neurological and psychiatric examination that the patient had speech latency of 20–30 seconds, dysarthria with slurred speech, impaired balance (not being able to stand on one foot at a time), slowness of motion, and appearing emotionally distanced.

The patient’s medical history, obtained from her parents, included uncomplicated mild asthma and allergy to pollen, without history of hospitalization. There is a family heredity for bipolar disorder I, but the patient had not had any prior history of psychiatric illness or drug abuse. Before admittance to the emergency department, the patient was in very good physical condition, being a top athlete in her field. The psychosocial family background of the patient was uncomplicated, as she grew up in a stable family, and attended school with good grades.

The patient’s confused thoughts, apathy, and difficulties talking were interpreted as psychotic symptoms. Risperidone was initiated. The patient’s condition deteriorated, and she began to display unrecognized signs of catatonia that included mutism, negativism, waxy flexibility, posturing, and difficulties with eating requiring a gastric feeding tube. After 2 weeks of treatment with risperidone with an orally administered dose of 1 mg twice daily and benzodiazepine in the form of midazolam with a dose of up to 7.5 mg three times daily, without any apparent positive effects, she developed deep vein thrombosis (DVT) due to immobilization. She was transferred to the university hospital for treatment. Upon renewed psychiatric assessment, her catatonic symptoms were recognized, applying the Appendix I section of the Bush–Francis Catatonia Rating Scale [7]. The patient responded positively on procedures 1, 2, 3, 4, and 7. Risperidone was discontinued, and lorazepam was readily initiated. The patient regained full orientation briefly. Nonetheless lorazepam 20 mg daily was not sufficient to achieve remission.

Olanzapine 2.5 mg daily was briefly tried. However, her general psychiatric condition continued to deteriorate, and she developed symptoms of waxy flexibility, posturing, stereotypes, derealization, and anxiety. She described a feeling of living in a dream, as if in “the Sims” game. Due to ongoing DVT treatment with anticoagulants, electroconvulsive therapy (ECT) was not considered an option.

Aripiprazole 7.5 mg per day was tested, and lorazepam was increased to 28 mg daily. Even if her condition initially improved, she eventually became increasingly catatonic. Antipsychotic treatment recurrently aggravated her symptoms and was discontinued altogether. Since remission was not achieved with lorazepam treatment alone and heredity for bipolar disorder was present in a first-degree relative, treatment with 126 mg lithium sulphate daily was initiated, which resulted in S-lithium concentration of 0.6 mmol/L. Concurrent treatment with lorazepam and adjunctive memantine with an orally administered dose of 10 mg daily, as suggested by Chaffkin *et al*. [8], was given.

After 2 weeks, she had improved enough to be able to leave the ward. Another week later the patient had fully recovered, 6 weeks after the initial presentation. No side effects were reported, and slow tapering of lorazepam and memantine was subsequently performed. Follow-up confirmed the continuous wellbeing with lithium as the sole medication.

### Investigations

Diagnostic workup performed during the patient’s initial hospitalization included urine toxicology screen, serum tests for ethanol, methanol, salicylate levels, creatinine, hepatic panel, and arterial blood gas, all of which were within normal limits. Nota bene (N.B.) that drug toxicology did not include “bath salts” or “ethyl chloride.” Cerebrospinal fluid (CSF) analyses revealed colorless and clear fluid at normal opening pressure with normal glucose, immunoglobin, lactate, and protein levels. The non-contrast head magnetic resonance imaging (MRI) scan of the brain was unremarkable, as was a routine electrocardiogram (EKG). A 20-minute video electroencephalogram (EEG) showed no evidence of underlying seizure disorder or focal neurologic dysfunction. Infectious etiologies were investigated with complete blood count, C-reactive protein (CRP), blood culture, urine culture, and analysis of cerebrospinal fluid for varicella and herpes. Secondary to immobilization in combination with a vascular variant (May–Thurner syndrome), the patient developed deep vein thrombosis. Ultrasound and phlebography of the left leg and pelvis in combination with computed tomography of the thorax showed a left-side ilio-femoral thrombosis that reached up in the vena cava inferior at the L4 spinal segment level.

### Discussion and conclusion

Unrecognized catatonic symptoms were first noticeable in our 17-year-old patient 1 week after presumed intake of the recreational designer drug “bath salts” and ethyl chloride. Consumption of bath salts/mephedrone has previously been associated with the onset of catatonia, as reported by Antunes *et al*. [9].

As antipsychotics have sometimes been used for catatonia, subsequent risperidone, olanzapine, and aripiprazole were tested to ameliorate the psychotic symptoms. However, this resulted in recurrent dose-dependent obvious deterioration in catatonic symptoms, which may be explained by the fact that dopamine D2 receptor blockade can sometimes worsen catatonic symptoms. Nonetheless, second-generation antipsychotics such as olanzapine and risperidone via 5HT2 receptor agonism is believed to stimulate dopamine release in relevant regions of the brain and to ameliorate catatonic symptoms [10]. In adolescents with catatonia exacerbated by psychotropic drugs, about half of the patients had a previous psychiatric medical history [11], and catatonia is sometimes the first recognized presentation of bipolar disorder [12]. This could possibly explain why the other individuals taking the same drugs did not develop catatonia. A lorazepam scheme was started with evident, but still limited, success. Lorazepam is a first line of treatment for catatonia, acting on inhibitory benzodiazepine receptors on the postsynaptic γ-aminobutyric acid (GABA)-A ligand-gated chloride channel.

In catatonia after drug withdrawal, treatment response may depend on the drug that induced it, and sometimes reinstitution might alleviate it. Since we were not certain of which drug the patient was exposed to and we had no further access to it, this approach was not readily available. Furthermore, ECT, which is another recommended line of treatment, was not possible due to ongoing anticoagulant treatment following a DVT. In theory, memantine may balance overactive NMDA signaling in lorazepam-resistant catatonia. Mephedrone, one of the constituents of “bath salt”, induces glutamatergic signaling. Memantine and lithium were added to normalize glutaminergic signaling and address an eventual underlying bipolar disease. Finally, a stable improvement was achieved, and 9 days later the patient was able to leave the hospital after 6 weeks of catatonia.

Withdrawal catatonia usually occurs after prolonged drug use, often months to years [13], in contrast to what happened in our patient, who became catatonic after only a few days of use. We therefore speculate that the patient had an inherited vulnerability to catatonia and/or that catatonia was the result of a malicious unrecognized consequence of newer dirty designer drugs, such as “bath salts”. Since memantine and lithium treatment was initiated simultaneously, we cannot know whether the final improvement was due to management of bipolar disorder and/or reconstitution of the receptor profile. However, the latter explanation of reconstitution of NMDA receptor signaling appears more likely given that lithium treatment usually takes weeks for effect, even if there are cases where a more rapid response has been reported [14]. The exact mechanism of lithium treatment is still unclear, even if neurotropic effects through inhibition of GSK-3-beta appear to be important. In our case the treatment response was rather rapid (9 days), and if lithium had an impact on the outcome in our case, it could possibly be due to decreased glutaminergic activation, which is another reported effect of lithium that could potentiate memantine [15]. The extent of effect and the exact mode of action of lithium in our case is uncertain. A spontaneous resolution unrelated to our treatment cannot be excluded but appears unlikely given the clinical course.

Irrespective of the mechanisms, the clinical implication of our case is that even a shorter drug exposure might trigger catatonia in selected patients.

## Data Availability

Not applicable
